# The Role of Persistent Inflammation in the Pathogenesis of Infected Nonunion: A Narrative Review

**DOI:** 10.1002/iid3.70260

**Published:** 2025-09-16

**Authors:** Tao Liu, Fengjiang Li, Yuanbin Yan, Silong Gao, Daqian Zhou, Xuanang Jiang, Chao Song, Zhijiang Fu, Zongchao Liu

**Affiliations:** ^1^ Luzhou Longmatan District People's Hospital Luzhou Sichuan Province China; ^2^ School of Integrated Traditional Chinese and Western Medicine Southwest Medical University Luzhou Sichuan Province; ^3^ Mianyang City College Mianyang Sichuan Province China; ^4^ Department of Orthopedics (Trauma and Bone‐setting), The Affiliated Hospital of Traditional Chinese Medicine Southwest Medical University Luzhou Sichuan Province China

**Keywords:** fracture healing, infectious bone disease, inflammation, osteogenesis

## Abstract

**Introduction:**

Infected Nonunion is a challenging condition that arises from infections at the fracture site, causing persistent inflammation and preventing proper healing of the fracture ends. This condition not only causes significant physical suffering but also imposes a heavy economic burden on patients. Despite the recognized link between Infected Nonunion and infection, the mechanisms underlying its occurrence and development remain incompletely understood. Recent studies have highlighted the close association between inflammatory factors and Infected Nonunion, suggesting that inflammation plays a pivotal role in its pathogenesis. However, a narrative review and summary of relevant literature are lacking. Therefore, this study aims to clarify the inflammatory mechanisms of Infected Nonunion and identify potential therapeutic targets.

**Methods:**

A comprehensive literature search was conducted to identify relevant domestic and international studies on the inflammatory mechanisms of Infected Nonunion. The search included databases such as PubMed using keywords related to Infected Nonunion, inflammation, and mechanisms. The selected studies were critically reviewed and summarized to extract key information on the inflammatory pathways, cytokines, and other relevant factors involved in Infected Nonunion.

**Results:**

The review identified several key inflammatory mechanisms that contribute to the development of Infected Nonunion. These include the activation of inflammatory cells, the release of inflammatory cytokines and chemokines, and the disruption of normal fracture healing processes.

**Conclusions:**

This narrative review elucidates novel perspectives on the inflammatory mechanisms in infected nonunion, with persistent inflammatory response triggered by pathogenic infection representing the core pathological process. The findings provide theoretical foundations for future research and therapeutic strategies, potentially facilitating the development of more effective treatments for infected nonunion. Targeted modulation of these inflammatory pathways may optimize fracture healing outcomes and alleviate the clinical burden of this condition.

## Graphical abstract

This paper summarizes recent literature on Infected Nonunion, a type of fracture nonunion caused by infection‐induced inflammation, highlighting the lack of understanding of its mechanisms despite its significant burden. Through a systematic review from an inflammatory perspective, the paper aims to provide new insights and theoretical support for future research and treatment.

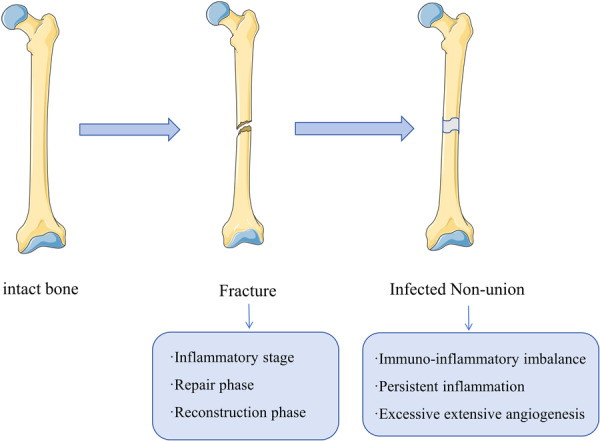

## 1 Introduction

Nonunion refers to a condition where a fracture has not healed within the expected timeframe (commonly 9 months) and remains unhealed even after an extended period of treatment (typically more than 3 months) [1]. Nonunion is one of the more common orthopedic complications in clinical practice, and it can occur in fracture patients of all age groups, with a higher incidence between the ages of 35 and 44. According to relevant data, the probability of nonunion varies with different fracture sites, with an overall incidence rate of 5% to 10% [1, 2, 3]. Infected Nonunion is a type of nonunion usually caused by an infection at the fracture site, leading to the fracture ends being unable to heal properly. Compared to simple nonunion, Infected Nonunion is one of the more challenging orthopedic diseases, typically caused by bacterial infections during or after surgery for open fractures, leading to an imbalance in the fracture healing mechanism. Its pathogenesis is complex, involving multiple mechanisms, including infection and fracture nonunion [4].

Currently, there are no specific medications for treating Infected Nonunion in clinical practice. The primary treatment method relies on thoroughly controlling the infection first, followed by a secondary fracture healing surgery according to standard fracture treatment protocols [5, 6]. This process usually requires long‐term treatment and rehabilitation, which not only brings significant psychological stress to patients but also imposes a heavy financial burden on them. Currently, the cost of treating nonunion is quite expensive. In the United States alone, each patient typically needs to spend tens of thousands of dollars, placing a heavy economic burden on both society and families [1, 7].

Currently, the academic community still lacks clarity on the mechanisms underlying the occurrence of Infected Nonunion. Some studies suggest that it is closely related to changes in the microenvironment, including the molecular aspects of bone infection necrosis, bone defects, and hematoma and soft tissue damage at the fracture site [5, 8]. Studying the mechanisms of Infected Nonunion from a microscopic perspective is of great significance.

**Figure 1:**
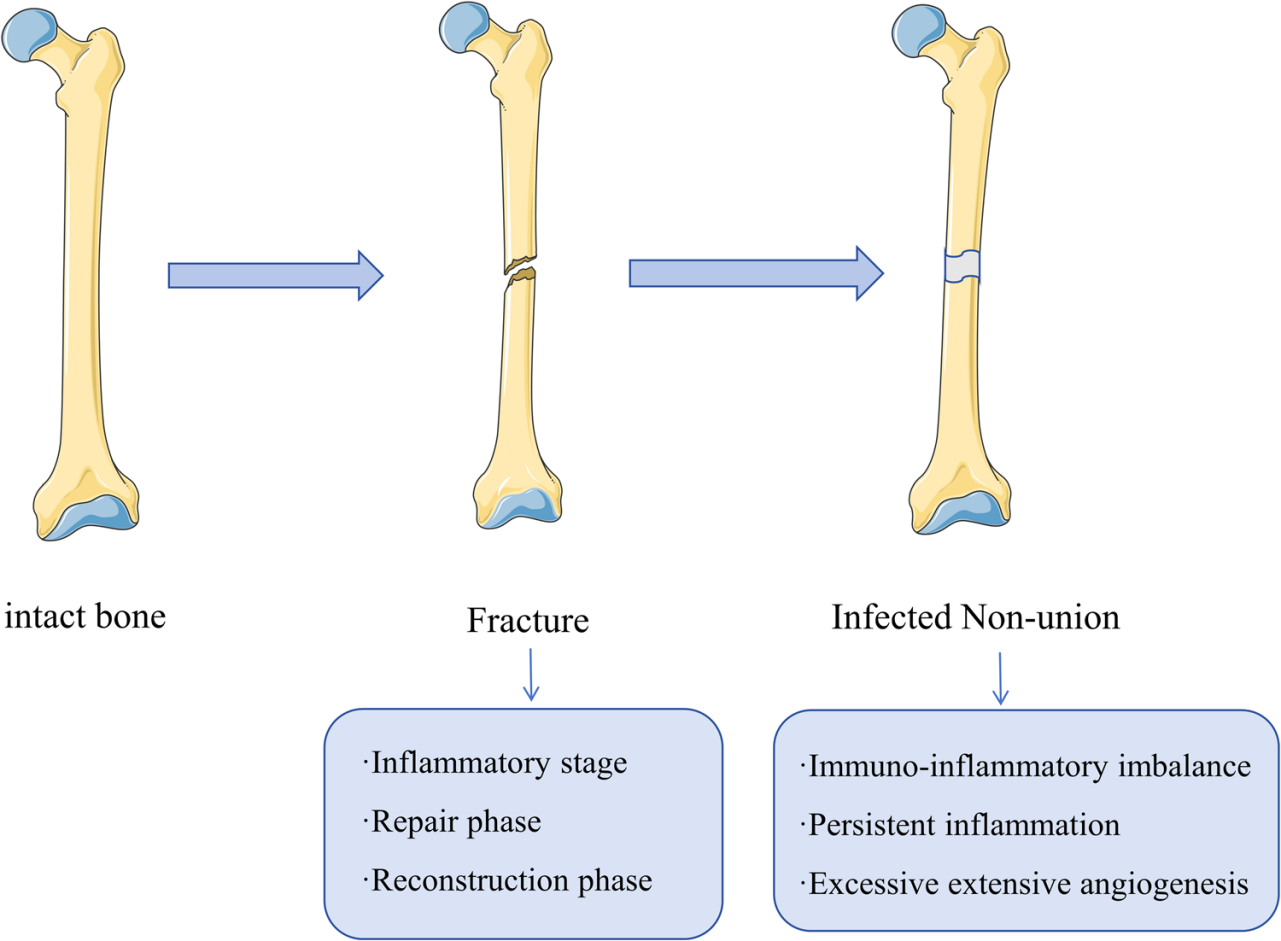
Normal bone fracture healing typically undergoes three stages, while infectious nonunion is often driven by inflammation as a critical pathogenic factor.

Research has found that the persistent inflammatory response triggered by the infection focus in Infected Nonunion is closely related to the hematoma at the fracture site and the condition of soft tissue damage [9]. The changes in the microenvironment caused by the persistent inflammatory response are closely related to the fracture healing process, the vascular disruption at the fracture ends, and the condition of the surrounding soft tissues [10]. Fracture hematoma plays a critical role in the bone healing process. However, impaired hematoma formation, premature clearance, or dysregulation of inflammatory‐coagulation signaling can disrupt the repair process, ultimately leading to nonunion [11, 12]. This risk is markedly increased under conditions of immune dysfunction or sustained aberrant inflammatory activation, further exacerbating the incidence of nonunion [11, 12]. These changes in the local microenvironment lead to the clinical difficulty in healing Infected Nonunion. This article will systematically reveal the microenvironmental changes caused by persistent inflammation during the occurrence of Infected Nonunion based on existing research findings. Furthermore, it will explore the potential mechanisms of Infected Nonunion healing to provide better guidance for clinical treatment (Figure 1).

## 2 Methods

This narrative review identified relevant studies published between 2000 and 2024 through PubMed and Web of Science searches. We included primary studies and meta‐analyses using keywords: “infected nonunion,” “bone infection,” “fracture healing,” and “inflammatory cytokines.” Only English‐language publications were considered. Exclusion criteria: non‐English studies. PRISMA guidelines were followed for methodology, and the ROBINS‐I tool was used to assess risk of bias in observational studies.

## 3 Fracture Healing Mechanism

Fracture healing is a complex biological process that involves multiple stages and various cell types. Generally, the process of fracture healing can be divided into three stages: the hematoma organization phase or inflammation phase, the primary callus formation phase or repair process, and the callus hardening and remodeling phase [2, 13].

### 3.1 Inflammatory Stage

Firstly, when a fracture occurs, the bone tissue, bone marrow, intramedullary blood vessels, and surrounding soft tissues at the fracture site are damaged. This leads to vascular dilation at the fracture ends and an inflammatory response characterized by the exudation of plasma and white blood cells [14]. The acute inflammatory response plays a crucial role in the fracture healing process. Within the first 24 h of a fracture, the damaged areas at the fracture ends and the surrounding soft tissues rapidly initiate an inflammatory response. In this process, neutrophils are among the first cells to be recruited to the fracture site. Through chemotaxis, they secrete pro‐inflammatory factors such as IL‐8, IL‐1, and TNF‐α at the fracture ends. These pro‐inflammatory factors not only enhance the chemotactic response of neutrophils but also promote local vascular dilation and increased permeability, allowing more inflammatory cells to enter the fracture site. Additionally, pro‐inflammatory factors activate and regulate the functions of other immune cells, further amplifying the inflammatory response [14, 15]. As the inflammation progresses, macrophages and lymphocytes are also attracted to the fracture site. Macrophages can phagocytize and clear necrotic tissue and cellular debris, creating favorable conditions for the subsequent repair process. Additionally, they secrete various growth factors and cytokines, such as insulin‐like growth factor‐I (IGF‐I), myostatin (also called growth differentiation factor‐8), members of the bone morphogenetic proteins (BMPs) family (including BMP‐2, BMP‐4, BMP‐5, BMP‐6), and osteonectin [13, 14]. Additionally, the damage at the fracture ends also causes local hypoxia and a decrease in pH levels. This activates thrombogenic factors and initiates angiogenesis. Various factors such as vascular endothelial growth factor (VEGF), basic fibroblast growth factor (bFGF), members of the transforming growth factor β (TGF‐β) family, hypoxia‐inducible factor (HIF), and angiopoietin‐1 are involved in the formation of new blood vessels and the construction of vascular networks. These processes ensure an adequate blood supply to the fracture site, facilitating the smooth progress of the repair process [14, 16, 17].

### 3.2 Repair Phase

Inflammatory cytokines play pivotal regulatory roles in bone remodeling. TNF‐α, a key pro‐inflammatory mediator, promotes mesenchymal stem cells (MSCs) migration to fracture sites and induces osteogenesis at low concentrations while inhibiting bone formation at high levels [18]. Additionally, it facilitates immune cell recruitment during early inflammation via the CCL2/CCR2 axis [18]. IL‐1, particularly IL‐1β, becomes activated during fracture healing, influencing bone metabolism by modulating osteoclast development while suppressing osteoblast proliferation and differentiation [18]. It also promotes MMP‐13 expression through the Wnt pathway, contributing to bone tissue remodeling [18]. IL‐6 regulates osteoblast and osteoclast activity, facilitating callus formation and angiogenesis during both acute and late phases of fracture repair [18]. Collectively, these pro‐inflammatory cytokines dynamically coordinate bone formation and resorption across different repair stages, maintaining the equilibrium of bone remodeling.

It is worth noting that the inflammation and neovascularization at the fracture ends secrete various growth factors and regulatory factors, such as transforming growth factor β (TGF‐β) and platelet‐derived growth factor (PDGF). These factors play an active and crucial role in regulating the bone repair process [19, 20]. They can promote the proliferation of cells from the bone marrow cavity, periosteum, surrounding tissues (such as muscles), and blood vessels (such as mesenchymal stem cells and fibroblasts), guiding these cells to migrate to the fracture site. Once these cells reach the fracture site, they participate in the formation of new bone and the repair of cartilage. For instance, TGF‐β and PDGF can enhance the proliferation and differentiation of mesenchymal stem cells, converting them into cells with osteogenic or chondrogenic capabilities, thereby boosting local osteogenic capacity [17, 19, 20, 21]. The enhancement of osteogenic capacity means accelerated bone formation activity. At the fracture ends, mesenchymal stem cells, under the stimulation of various growth factors such as TGF‐α2, PDGF, IGF‐1, and BMPs (including BMP‐2, BMP‐4, BMP‐5, BMP‐6), proliferate and differentiate into chondrocytes. These chondrocytes subsequently form a cartilage callus, providing initial mechanical stability to the fracture site [22].

Interestingly, the local osteoclast activity is greatly inhibited at this stage. For example, osteoprotegerin (OPG) regulates bone metabolism by inhibiting osteoclast differentiation and activity through blockade of the RANKL–RANK signaling pathway. Additionally, signals such as SEMA3A–NRP1 secreted by osteoblasts, along with anti‐inflammatory cytokines like IL‐4 and IL‐10, also suppress RANKL‐mediated osteoclastogenesis, thereby contributing to the maintenance of bone metabolic homeostasis [23, 24, 25]. Osteoclasts are primarily responsible for bone resorption and remodeling, but during the initial bone callus formation phase, their activity is suppressed to ensure the stable growth of the bone callus at the fracture ends [16]. As the healing process progresses, osteoblasts begin to synthesize intramembranous woven bone at the distal fracture site. This woven bone gradually replaces the cartilage through endochondral ossification, forming a hard callus [26]. However, this process is relatively slow, with the initial healing of the fracture typically completed within approximately 6 to 10 weeks post‐injury.

### 3.3 Reconstruction Phase

The formation of the callus is a crucial step in fracture healing. It is a temporary structure formed by newly generated bone tissue that provides the necessary support and stability to the fracture site. However, to restore the bone at the fracture ends to its original state, the callus must undergo mineralization and remodeling to return to a state similar in shape, size, and biomechanical capability to the original bone [26]During this process, osteoblasts begin to secrete signaling molecules such as BMPs and Wnt signaling molecules. These molecules stimulate osteoblasts to secrete bone matrix, which gradually calcifies. This bone matrix subsequently undergoes mineralization, forming new bone tissue [27].

Bone mass changes are also influenced by cytokines and growth factors secreted by osteocytes, such as osteoprotegerin (OPG, a member of the TNF family) and receptor activator of nuclear factor κB ligand (RANKL), which regulate osteoclast activity. Naturally, when the new bone tissue forms and reaches a certain strength and stability, the bone remodeling process will terminate on its own [28]. Additionally, osteocytes can control the balance between bone resorption and formation through the Wnt signaling pathway, thereby ensuring the stability and integrity of the skeletal structure [28]. Naturally, the remodeling of a fracture is already a later stage in the healing process. However, complete recovery is a complex and lengthy process. Only when the original structure of the bone, including both cortical and cancellous bone, is restored can the fracture site regain sufficient strength and stability [2]. Throughout the entire fracture healing process, these three steps follow a sequential order. However, these stages are interrelated. Understanding the three periods of normal fracture healing helps us grasp the mechanisms of infected nonunion, thereby revealing the intricacies of infected nonunion from a localized and detailed perspective.

## 4 Infected Nonunion

### 4.1 Immuno‐Inflammatory Imbalance

Infected nonunion is a severe complication of fracture‐related infection (FRI) [29]. It is clinically defined as a fracture that fails to heal after 9 months of treatment with no evidence of progressive bone healing for at least 3 consecutive months in the presence of persistent FRI at the fracture site. When the fracture ends are infected by pathogens, particularly bacteria such as *Staphylococcus aureus*, it leads to inflammation, necrosis of the surrounding soft tissue, and obstruction of new bone formation around the fracture site. This results in delayed healing or non‐healing of the fracture, known as infected nonunion [20, 30, 31, 32, 33]. There are various types of bacteria at the infection site of infected nonunion, with *S. aureus* being one of the primary bacteria associated with it. Clinical treatment typically requires extensive use of antibiotics, but these pathogens often form biofilms, allowing them to survive antibiotic treatment. This is one of the reasons why the infection is difficult to control [33, 34]. Although the presence of these bacteria activates the body's immune response and causes an acute inflammatory reaction, it can aid the body's self‐repair process [14]. However, if the infection site cannot be effectively controlled, the inflammatory response will persist. Since infected nonunion is inevitably associated with this ongoing inflammation, once infected nonunion occurs, it is often chronic and has a prolonged course [2, 35]. Currently, we have found that the presence of infectious necrotic foci in infected nonunion can produce various virulence factors, such as toxins and enzymes. Studies have shown that in infectious nonunion, *S. aureus* induces osteoblast death by secreting membrane‐disrupting virulence factors, such as toxins and enzymes [35, 36]. This process is characterized by non‐apoptotic forms of cell death in osteoblasts, including necroptosis and pyroptosis. The loss of osteoblasts not only directly impairs bone formation capacity but also contributes to compromised bone healing and exacerbates the pathological progression of infectious nonunion [35, 36]. Immune cells within human tissues, such as macrophages, dendritic cells, and lymphocytes, respond quickly and recognize the invasion. Once pathogens or damage are detected, these cells release various pro‐inflammatory mediators, including IL‐1β, TNF‐α and chemokines [37, 38]. As previously mentioned, these mediators can initiate and regulate the inflammatory response, attracting more immune cells such as polymorphonuclear neutrophils (PMNs), monocytes/macrophages, and lymphocytes to be activated and infiltrate the infection site or damaged area. During the inflammatory response in the normal fracture healing stage, M1 phenotype macrophages secrete pro‐inflammatory cytokines like TNF‐α and IL‐1β to promote inflammation. However, as the inflammation persists and expands, macrophages undergo a transition from the M1 phenotype to the M2 phenotype [39, 40]. M2 phenotype macrophages can secrete anti‐inflammatory cytokines such as IL‐10 family cytokines and TGF‐β. These cytokines can inhibit the inflammatory response and promote tissue repair. For example, IL‐10 family cytokines can activate the JAK/STAT signaling pathway to regulate inflammation [40, 41, 42]. Additionally, IL‐10 can promote the M2 phenotype of macrophages, which in turn can enhance angiogenesis and tissue repair [43, 44, 45]. TGF‐β can activate the SMAD signaling pathway, which promotes the differentiation of fibroblasts and endothelial cells, further aiding in tissue repair and reconstruction [46]. Additionally, TGF‐β can regulate the activation and proliferation of T cells, thereby modulating the inflammatory response [47]. Therefore, if macrophages cannot effectively transition from the pro‐inflammatory M1 phenotype to the anti‐inflammatory tissue‐repairing M2 phenotype after a fracture, the bone tissue repair process may be hindered [48]. However, in cases of infectious nonunion of bones, the persistent presence of infection and the production of pro‐inflammatory cytokines disrupt the adaptive balance within the body. Macrophages are unable to effectively transition from the pro‐inflammatory M1 phenotype to the anti‐inflammatory, tissue‐repairing M2 phenotype. Consequently, the imbalance in the M1/M2 macrophage ratio, where the production of pro‐inflammatory cytokines is not effectively controlled, leads to a sustained and expanded inflammatory response [40, 48]. Interestingly, under the influence of the local microenvironment, inflammatory cytokines can have different effects under various physiological or pathological conditions. Previously, we discussed that in the regulation of inflammation, the osteogenic differentiation of MSCs can be influenced by various cytokines secreted during inflammation. Among these, important factors such as IL‐1β and TNF‐α play a significant role in bone tissue repair. They can affect the differentiation of mesenchymal stem cells and participate in the repair and reconstruction of bone tissue. However, recent research indicates that the concentration and duration of these cytokines have a significant impact on the osteogenic differentiation of mesenchymal stem cells [49]. Therefore, an excessive inflammatory response can lead to tissue damage and bone resorption at the fracture ends in infectious nonunion. Such a response may inhibit the differentiation process of osteoblasts, becoming a key reason for bone non‐union. For instance, under the influence of chronic inflammatory factors, a large release of pro‐inflammatory cytokines such as IL‐1, IL‐6, and TNF occurs [50, 51]. These cytokines stimulate osteoblasts and activated T cells to release RANKL. This protein interacts with RANK on the surface of osteoclasts, leading to their excessive activation, greatly enhancing the bone resorption capacity of osteoclasts, thus accelerating bone loss and destruction [14, 49, 50, 52, 53]. In conclusion, persistent inflammation induced by infectious foci constitutes a major contributing factor to the development of infected nonunion.

### 4.2 Persistent Inflammation and Bone Repair

Bone defects are also a significant factor in infectious nonunion. These defects compromise the stability of the fracture ends, making bone healing more difficult [31, 54]. Clinically, acquired bone defects are mainly caused by trauma, leading to direct bone loss. Additionally, open fractures may result in bone defects because local bone fragments can fall into surrounding spaces, creating defects. In closed fractures, bone defects may be caused by handling or abnormal activities that lead to wear at the fracture ends. These worn fragments are absorbed by the surrounding tissues, which in turn causes non‐union of the fracture ends [31, 54]. At the fracture site, MSCs in the bone marrow play a crucial role in repair. They are regulated by various growth factors and signaling molecules, such as BMPs, TGF‐β, and fibroblast growth factor (FGF). These growth factors participate in bone formation by stimulating the differentiation, proliferation, and activity of osteoblasts, thereby promoting bone tissue regeneration and repair [55, 56].

Among these, bone morphogenetic proteins (BMPs) are unique cytokines with osteoinductive activity. They can irreversibly induce the differentiation of mesenchymal cells into chondrocytes and osteoblasts. BMPs also promote the formation of osteoblasts and the deposition of bone matrix, thereby facilitating the formation of new bone [57]. Specifically, BMPs bind to their receptors and primarily activate a series of signaling pathways, including the BMP signaling pathway, the Smad pathway, and the MAPK pathway. These signaling pathways regulate the proliferation, differentiation, and function of osteoblasts, thereby influencing bone formation and repair [58, 59]. Additionally, BMPs can stimulate the mineralization of osteoblasts, promoting the maturation and stabilization of bone. Besides their direct effects on mesenchymal stem cells, BMPs can influence other cells related to bone formation, such as fibroblasts and vascular endothelial cells. Under the induction of BMPs, these cells can differentiate into osteoblasts or form blood vessels, both of which are essential for fracture repair. This further contributes to bone formation and repair [60]. However, when chronic inflammation is excessive or persistent, it can inhibit the action of BMPs. Inflammatory cells can produce inflammatory mediators, such as prostaglandin E2 (PGE2), which can bind to BMP receptors and thereby inhibit BMP signaling [61, 62]. Additionally, persistent inflammatory responses can induce the expression of BMP antagonists, such as Noggin and Gremlin. These antagonists can bind to BMP ligands, thereby inhibiting the effects of BMPs [63, 64]. Inflammatory responses can activate the NF‐κB signaling pathway, which in turn inhibits the BMP signaling pathway, affecting the growth and differentiation of periosteal cells [65]. Moreover, TGF‐β induced by inflammatory responses can cross‐talk with Smad proteins in the BMP signaling pathway, exerting a negative effect on fracture repair [66]. These are all important reasons for non‐union of fractures.

Additionally, certain cytokines that normally play critical regulatory roles in fracture repair, such as transforming growth factor (TGF‐β1), insulin‐like growth factor (IGF), and FGF, become less effective due to persistent inflammation caused by infection, making it difficult for them to function properly in cases of infectious non‐union of fractures [67, 68, 69]. Transforming growth factor‐β (TGF‐β) plays a complex role in skeletal development and maintenance, particularly in the differentiation and maturation of osteoblasts and osteoclasts. TGF‐β1 can stimulate the proliferation and differentiation of fibroblasts, endothelial cells, and bone marrow mesenchymal stem cells, promoting the formation of granulation tissue, fibrous tissue, and bone tissue. However, in the later stages of these cells' maturation, its effects may become less pronounced or even inhibitory [70]. Studies have found that persistent inflammatory responses increase the levels of cytokines such as TGF‐β1, which can impair BMSC‐mediated bone regeneration. Excessive TGF‐β1 induces apoptosis through the TGF‐β/Smad3 signaling pathway by upregulating apoptotic genes like Bax and Bad. Additionally, it inhibits the proliferation and differentiation of osteoblasts by suppressing runt‐related transcription factor 2 (Runx2) [71]. IGF plays a crucial role in the fracture healing process. IGF can promote the proliferation and differentiation of osteoblasts, facilitating the formation and mineralization of bone tissue. In cases of fracture site defects, the expression levels of IGF may increase to stimulate healing at the fracture site and aid in the regeneration and repair of skeletal muscle [72]. FGF not only stimulates the formation of new blood vessels and increases blood circulation at the fracture site, providing the necessary nutrients and oxygen for healing, but also promotes the proliferation and differentiation of osteoblasts, aiding in the repair and regeneration of bone tissue [73]. However, under the influence of persistent inflammation in infected nonunion, The bFGF level is abnormal, leading to non‐union of the bone [74]. For example, Chen QQ and others have found that reduced levels of FGF‐2 and IGF‐1 in a diabetic rat model lead to non‐union of the bone [75]. Its family members, such as FGF‐2 (also known as basic fibroblast growth factor, bFGF), are supposed to participate in the signaling transduction of periosteal growth factors, thereby affecting fracture healing [76]. Additionally, persistent inflammation can regulate the expression of FGF23. The increased expression of FGF23 is associated with renal osteodystrophy and abnormal bone metabolism, which may also be a key factor in the formation of nonunion. Persistent inflammation will activate hypoxia‐inducible factor (HIF)−1α, thereby stimulating the expression of FGF23. The excessive expression of FGF23 will interfere with bone mineralization, which is an important factor in the occurrence and development of infected nonunion [77, 78, 79]. Under persistent inflammation, the mechanisms of action of the aforementioned cytokines also change, which is detrimental to fracture healing. These changes are also one of the important reasons for the occurrence of infected nonunion.

### 4.3 Excessive Extensive Angiogenesis

Bone is a highly vascularized tissue. Blood vessels in bone tissue provide the necessary nutrients and oxygen to the bone, while also removing metabolic waste and carbon dioxide from the body. During fracture healing, the formation of new blood vessels is crucial for the repair of the fracture. New bone needs nutrients from the blood to grow, and the newly formed blood vessels can provide the necessary nutrients and oxygen to the fracture site [80]. The current academic consensus is that if blood vessels are damaged and the blood supply is insufficient, the fracture healing process may be delayed or inhibited. At the same time, osteoblasts form a callus at the fracture site and transport large amounts of minerals such as calcium and phosphate ions through the blood vessels to promote bone healing. Interestingly, excessive angiogenesis and vascularization may also hinder adequate fracture repair and lead to the formation of nonunion [81].

Under normal circumstances, during the inflammatory response in the fracture healing process, inflammatory cytokines can promote the transcription and expression of the VEGF gene by activating the STAT3 and NF‐κB signaling pathways within cells [80]. VEGF is a potent proangiogenic factor that stimulates the proliferation, migration, and formation of new vascular networks of endothelial cells. By stimulating the expression of VEGF, these inflammatory cytokines can further promote angiogenesis and blood supply at the fracture site, providing essential repair conditions such as nutrients and oxygen for fracture healing [80].

**Figure 2:**
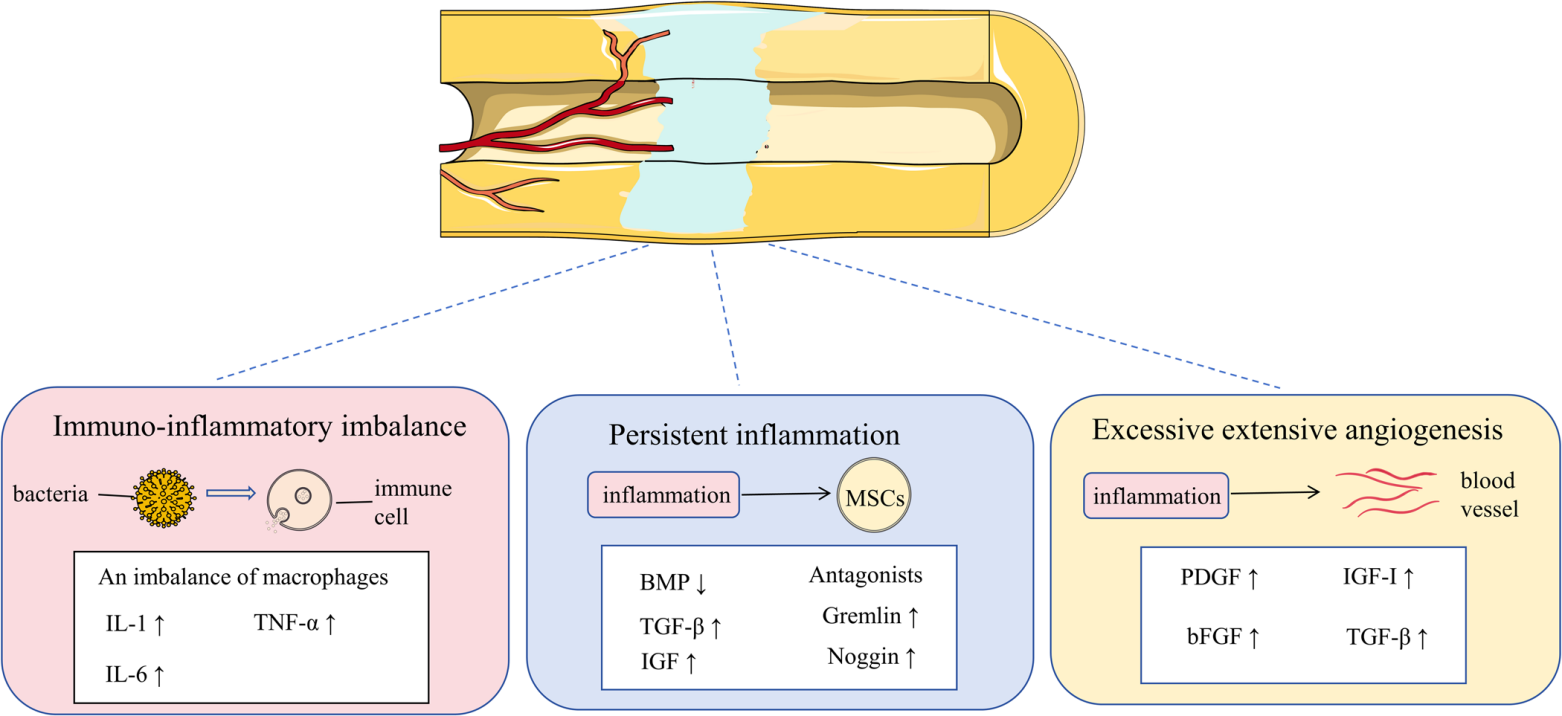
Pathogenesis of persistent inflammation in infectious nonunion.

However, the fracture ends in infected nonunion often exhibit excessive angiogenesis and vascularization. This occurs because inflammatory mediators are released and accumulate at the site of infection, recruiting and activating pro‐inflammatory cells. Under this stimulation, angiogenesis becomes excessive and abnormal. This excessive angiogenesis and vascularization become one of the significant obstacles to the healing of infected nonunion [81]. The excessive formation of blood vessels in infected nonunion is induced by IL‐6 secreted by macrophages and T cells, which can upregulate VEGF expression via the JAK2/STAT3 signaling pathway. High levels of VEGF further activate the VEGF/VEGFR signaling pathway, promoting additional angiogenesis and increasing vascularization at the fracture ends [81, 82, 83]. VEGF, as a growth factor specifically acting on vascular endothelial cells, promotes vascular development by binding to VEGFR. The two main types of VEGFR are VEGFR‐1 (Flt‐1) and VEGFR‐2 (KDR/Flk‐1). When VEGF binds to these receptors, it activates them and triggers intracellular signaling pathways, making a significant contribution to early vascular formation [84]. Under the stimulation of inflammation in infected nonunion, the VEGF/VEGFR signaling pathway becomes excessively activated [85]. This excessive activation stimulates endothelial cells to proliferate and migrate, leading to the formation of more blood vessels [84]. Platelet‐derived growth factor (PDGF) is also one of the important cytokines promoting angiogenesis. It is a factor that can stimulate the activation of fibroblasts and mesenchymal stem cells. PDGF‐BB binds to its receptor (PDGFRββ), stimulating the chemotaxis of fibroblasts and promoting angiogenesis [86, 87, 88]. In addition, inflammatory secreted factors such as basic bFGF, IGF‐I, and TGF‐βare all important cytokines promoting angiogenesis [89]. Moreover, IGF, FGF, and TGF can also promote angiogenesis and tissue regeneration, influencing multiple stages of angiogenesis [81, 90, 91]. In conclusion, although proper angiogenesis and vascularization are necessary in the early stages of fracture repair, the excessive release of these proangiogenic factors under sustained inflammatory responses makes it difficult to balance the extent of angiogenesis and vascularization. This excessive angiogenesis and vascularization hinder fracture repair [81] (Figure 2).

## 5 Discussion

**Figure 3:**
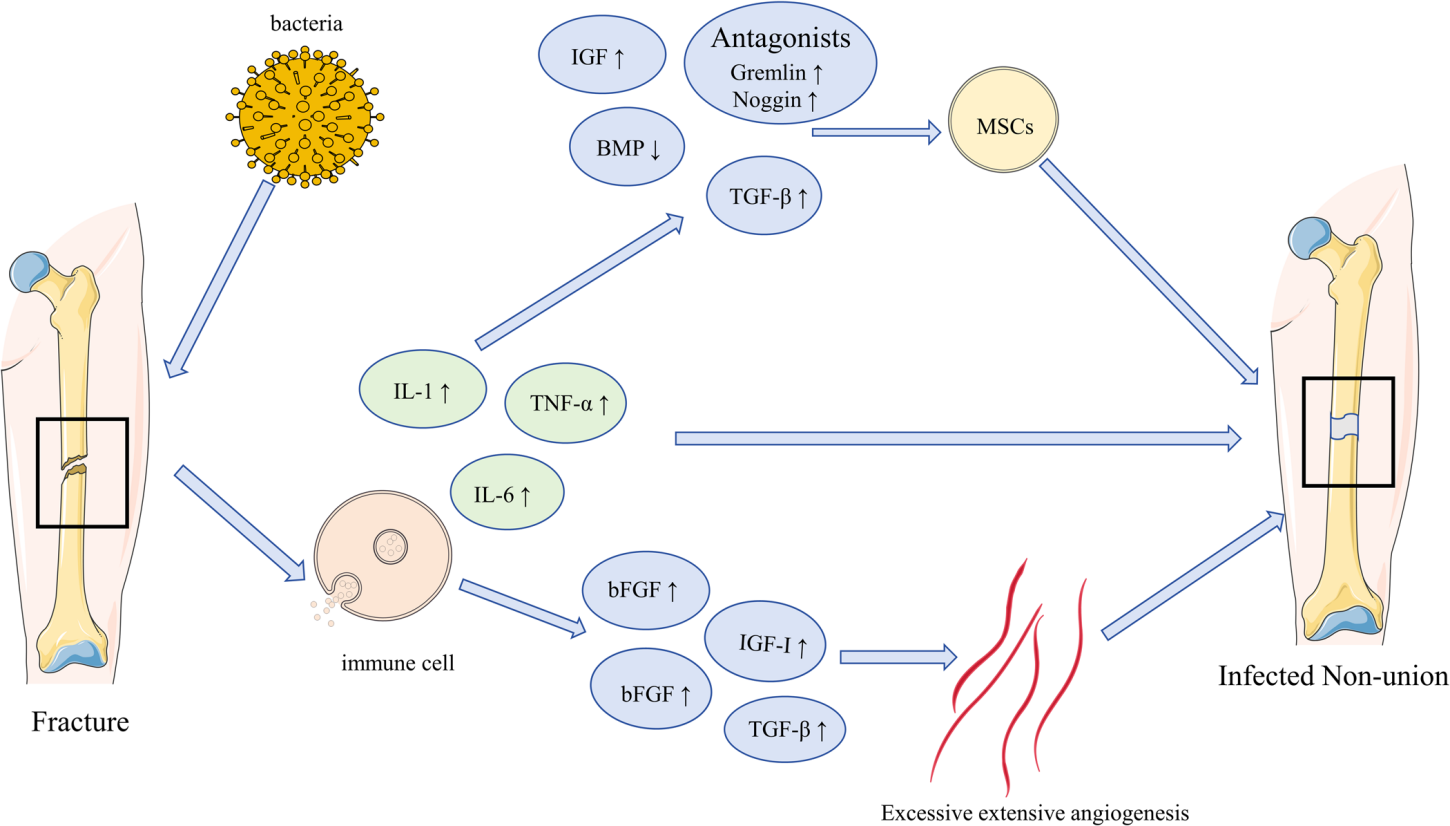
Specific molecular pathogenesis of infectious nonunion under the influence of inflammation.

The chronic infection source in infected nonunion induces persistent inflammation, which hinders the repair and remodeling of bone tissue. Inflammation has a dual role in fracture healing: while a moderate amount of inflammation aids bone repair, the chronic inflammation caused by infected nonunion leads to an excessive inflammatory response that delays or prevents the healing of fracture ends. Anti‐inflammatory drugs are commonly used to reduce or eliminate inflammatory responses in the body. In orthopedic practice, particularly in the management of fracture‐related pain, nonsteroidal anti‐inflammatory drugs (NSAIDs) are widely prescribed [92]. Existing evidence suggests that short‐term, low‐dose administration of anti‐inflammatory agents—such as NSAIDs or selective cyclooxygenase‐2 (COX‐2) inhibitors—is relatively safe for postoperative analgesia [92, 93]. However, prolonged or high‐dose use may impair bone healing by inhibiting prostaglandin synthesis, thereby increasing the risk of nonunion [92, 93, 94, 95]. Furthermore, patients treated with NSAIDs have been shown to exhibit a higher incidence of complications, including nonunion, malunion, and infection [96]. Therefore, it is necessary to elucidate the precise mechanisms of infected nonunion development from an inflammatory perspective, based on detailed evidence. Moreover, the critical role of inflammatory biomarkers in such conditions warrants emphasis. Substantial evidence demonstrates that inflammatory markers serve as reliable predictors for complications associated with open fractures, including infection, osteomyelitis, and nonunion. These biomarkers can function as early screening tools for infection, enabling timely clinical intervention [97, 98]. Regenerative medicine offers innovative solutions for bone repair by integrating cell therapy, biomaterials, and bioactive molecules in synergistic strategies that dynamically regulate the balance between the bone immune microenvironment and osteogenesis [99, 100]. In previous work, our research team also achieved spatiotemporal coordination of inflammation modulation and bone regeneration through an intelligent drug delivery system [100]. Using microfluidic technology, we developed a composite hydrogel material that can suppress inflammation in the early stage and promote osteogenesis in the later stage [100]. By precisely regulating the bone immune microenvironment, this approach significantly enhances repair efficiency and provides new perspectives for the clinical treatment of bone defects [100]. Our research team will continue to study and validate specific measures and strategies for treating infected nonunion in the future (Figure 3).

## 6 Conclusion

This narrative review elucidates novel perspectives on the inflammatory mechanisms in infected nonunion, with persistent inflammatory response triggered by pathogenic infection representing the core pathological process. The findings provide theoretical foundations for future research and therapeutic strategies, potentially facilitating the development of more effective treatments for infected nonunion. Targeted modulation of these inflammatory pathways may optimize fracture healing outcomes and alleviate the clinical burden of this condition.

## Author Contributions

**Tao Liu:** writing – original draft. **Fengjiang Li:** visualization. **Yuanbin Yan:** software. **Silong Gao:** writing – original draft. **Daqian Zhou:** resources. **Xuanang Jiang:** software. **Chao Song:** software, supervision. **Zhijiang Fu:** writing – review and editing.

## Ethics Statement

The authors have nothing to report.

## Conflicts of Interest

The authors declare no conflicts of interest.

## Data Availability

The authors have nothing to report.
